# The Immunomodulatory Effects of Vitamin D on COVID-19 Induced Glioblastoma Recurrence via the PI3K-AKT Signaling Pathway

**DOI:** 10.3390/ijms252312952

**Published:** 2024-12-02

**Authors:** Bi-Tian Zhang, Ping-Chung Leung, Chun-Kwok Wong, Dong-Jie Wang

**Affiliations:** 1 Institute of Chinese Medicine, State Key Laboratory of Research on Bioactivities and Clinical Applications of Medicinal Plants, The Chinese University of Hong Kong, Hong Kong, China; bitianzhang@link.cuhk.edu.hk (B.-T.Z.); pingcleung@cuhk.edu.hk (P.-C.L.); 2 Department of Chemical Pathology, The Chinese University of Hong Kong, Prince of Wales Hospital, Hong Kong, China; 3 Li Dak Sum Yip Yio Chin R & D Centre for Chinese Medicine, The Chinese University of Hong Kong, Hong Kong, China

**Keywords:** COVID-19 induced glioblastoma recurrence, vitamin D, PI3K-AKT signaling pathway, pharmacology network

## Abstract

Glioma is a highly invasive brain cancer that is difficult to treat due to its complex molecular characteristics and poor prognosis. The COVID-19 pandemic has introduced additional clinical challenges for cancer patients, especially those with glioma. This study explored the molecular interactions between glioma and COVID-19 using integrated bioinformatics methods, including enrichment analysis, survival analysis, and molecular docking, focusing on the PI3K-Akt signaling pathway and the immunomodulatory role of vitamin D. From gene expression data of glioma and COVID-19, 203 common differentially expressed genes were identified, and six prognostic key genes—MYBL2, RBM6, VEPH1, AHNAK2, GNG10, and DUSP14—were further determined. After intersecting with vitamin D targets five prognostic key genes were determined—MYBL2, RBM6, VEPH1, AHNAK2 and GNG10. These genes play significant roles in the PI3K-Akt pathway and potentially interact with vitamin D. Molecular docking and single-cell RNA sequencing analyses suggest that vitamin D may improve the prognosis of glioma patients infected with COVID-19 by regulating these key genes and the PI3K-Akt pathway. The findings reveal molecular links between glioma and COVID-19, thereby providing new insights for developing targeted therapeutic strategies.

## 1. Introduction

Glioma, a heterogeneous and highly aggressive form of brain cancer, represents one of the most challenging malignancies to treat due to its complex molecular landscape and poor prognosis [1]. Despite advancements in surgical techniques, radiation therapy, and chemotherapy, the median survival time for patients with high-grade gliomas remains dismal [2], underscoring the urgent need for novel therapeutic targets and prognostic biomarkers. Concurrently, the global outbreak of Coronavirus Disease 2019 (COVID-19), caused by the severe acute respiratory syndrome coronavirus 2 (SARS-CoV-2), has posed significant challenges to healthcare systems worldwide, particularly affecting vulnerable populations, including cancer patients [3]. Emerging evidence suggests that COVID-19 may exacerbate the clinical course of cancer, including glioma [4], by modulating immune responses and influencing tumor biology. The recurrence of glioma presents significant challenges in clinical oncology, and the advent of the COVID-19 pandemic has introduced additional complexities that may influence tumor behavior and patient outcomes. Despite extensive research on glioma and COVID-19 individually, the intersection of these two conditions remains largely unexplored, representing a critical gap in our understanding of cancer progression in the context of infectious diseases. The intersection of glioma pathogenesis and COVID-19-induced molecular alterations warrants comprehensive investigation to elucidate shared pathogenic mechanisms and identify potential therapeutic targets [4]. Understanding the interplay between these two conditions is critical, especially considering the immunosuppressive environment often present in glioma patients [5], which may predispose them to more severe outcomes upon SARS-CoV-2 infection [6].

Central to both glioma progression and viral infections is the phosphatidylinositol 3-kinase (PI3K)-Akt signaling pathway, a pivotal regulator of cellular processes including growth, proliferation, survival, and metabolism. The PI3K signaling pathway is known for its role in cellular processes such as growth, proliferation, and survival [7]. Dysregulation of the PI3K-Akt pathway has been implicated in oncogenesis [8], tumor progression [9], and resistance to therapy in various cancers, including glioma [10]. This pathway also plays a significant role in the host response to viral infections, influencing viral replication and immune modulation [11,12]. The prominence of the PI3K pathway in both diseases highlights its potential as a therapeutic target. The convergence of PI3K-Akt signaling in the context of glioma and COVID-19 highlights its potential as a therapeutic nexus for mitigating the adverse effects of SARS-CoV-2 on glioma outcomes.

Vitamin D, a secosteroid hormone, has garnered attention for its immunomodulatory and anti-inflammatory properties [13], which may influence both cancer progression and the immune response to viral infections [14]. Recent studies suggest that adequate levels of Vitamin D may confer protective effects against severe COVID-19 [15] and potentially modulate tumor growth and immune evasion in glioma [16]. Investigating the role of vitamin D in the molecular interplay between COVID-19 and glioma could unveil novel strategies for enhancing patient prognosis and resilience against concurrent pathological insults [17].

In this study, we employed comprehensive bioinformatics approaches, including enrichment analysis, survival analysis, and molecular docking, to identify and characterize key genes that are differentially expressed in both glioma and COVID-19 contexts. Employing bioinformatics approaches is essential in this investigation, as they enable the integration and analysis of large-scale genomic and clinical datasets to uncover potential molecular mechanisms linking COVID-19 infection with glioma recurrence. Bioinformatics tools such as enrichment analysis, survival analysis, and molecular docking facilitate the identification of key genes and pathways that may be pivotal in mediating the interplay between these diseases. This study aims to address this unexplored area by leveraging computational methods to identify shared genetic factors and potential therapeutic targets, thereby providing insights that could inform personalized treatment strategies and improve prognostic outcomes for glioma patients amidst the ongoing pandemic. The relevance of this work lies in its potential to bridge the knowledge gap between oncological and infectious disease research, offering a foundation for more comprehensive patient management and the development of innovative therapeutic interventions.

In summary, this study provided a comprehensive analysis of the crosstalk between glioma and COVID-19 at the genetic level, emphasizing the significance of the PI3K-Akt pathway and vitamin D-mediated immunomodulation. The findings not only contribute to our understanding of these diseases but also pave the way for future research aimed at developing novel interventions to improve outcomes for glioma patients affected by COVID-19.

## 2. Results

### 2.1. Enrichment Analysis of Common Gene Targets Between Glioma and COVID-19 Differentially Expressed Genes

**Figure 1:**
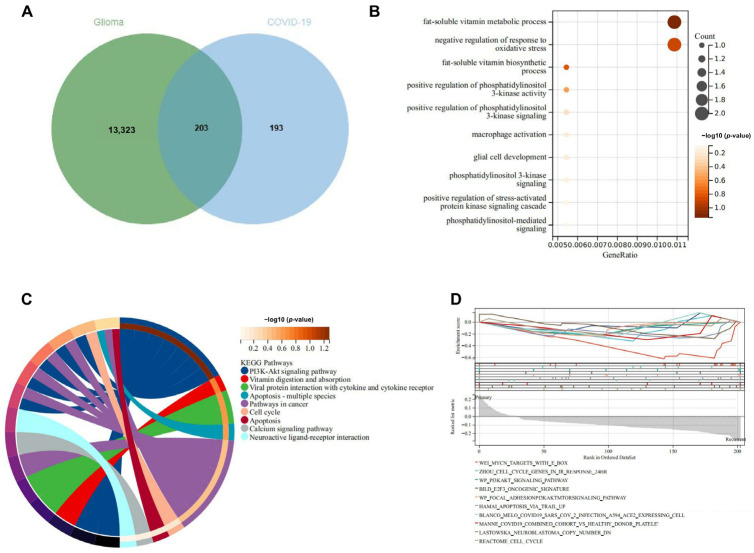
This figure presented the enrichment analysis of significant differential genes intersecting in glioma and COVID-19, focusing on the PI3K-Akt signaling pathway. (**A**) Venn diagram illustrating the overlap of differentially expressed genes between glioma and COVID-19 datasets, with 203 genes identified as common. (**B**) Biological process enrichment analysis revealed significant pathways including phosphatidylinositol 3-kinase (PI3K) signaling, highlighting its role in cellular processes such as fat-soluble vitamin biosynthesis and oxidative stress regulation. (**C**) The KEGG pathway analysis using a chord diagram highlighted the involvement of intersecting genes in pathways such as the PI3K-Akt signaling pathway, which is crucial for cancer-related processes and the immune response. (**D**) GSEA pathway analysis underscored the enrichment of the PI3K-Akt signaling pathway, among other pathways, indicating its significant contribution to the pathological mechanisms shared by glioma and COVID-19. This comprehensive analysis underscored the critical role of the PI3K-Akt signaling pathway in the intersection of pathways involved in both diseases, providing insights into potential therapeutic targets.

The presented figure illustrates the results of an enrichment analysis focusing on overlapping differentially expressed genes associated with glioma (Supplement Table S1) and COVID-19 (Supplement Table S2). The Venn diagram in the Figure 1A depicted a significant intersection, highlighting 203 shared genes between glioma and COVID-19. Following this, Figure 1B showcased the biological processes enriched within these overlapping genes, with particular emphasis on pathways related to the phosphatidylinositol 3-kinase (PI3K) signaling pathway, which is notably involved in cellular functions such as growth, proliferation, and survival.

In Figure 1C, a Chord diagram demonstrated the KEGG pathways affected by the intersecting genes, again underscoring the PI3K-Akt signaling pathway as a critical player in the molecular landscape of both conditions. This figure presented a comprehensive overview of the intricate relationships among the various pathways, illustrating the pathways implicated in tumorigenesis and viral infection. Finally, Figure 1D provided a Gene Set Enrichment Analysis (GSEA) plot, revealing the enrichment scores for various pathways, thereby affirming the prominent role of the PI3K pathway in the context of both glioma and COVID-19 pathophysiology.

### 2.2. Screening of Key Genes Involved in COVID-19 Induced Glioma

**Figure 2:**
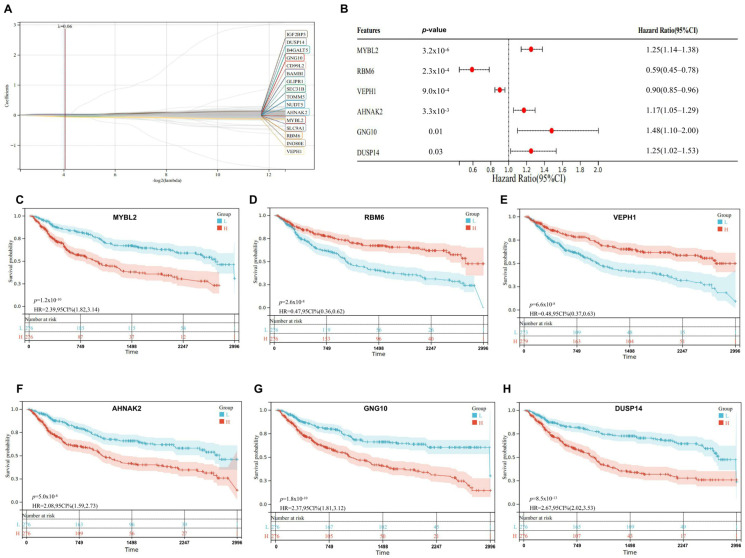
This figure illustrated the selection and analysis of significant differential genes related to COVID-19-induced glioma using LASSO-Cox regression and forest plot methodology. (**A**) The LASSO coefficient profile plot identified optimal gene features by tuning lambda parameters. (**B**) The forest plot presented the selected genes, their *p*-values, and hazard ratios (HR) with 95% confidence intervals (CI), indicating their association with survival outcomes in glioma patients. (**C**–**H**) Kaplan–Meier survival curves for each significant gene (MYBL2, RBM6, VEPH1, AHNAK2, GNG10, DUSP14) depicted the survival probability over time for two patient groups (high vs. low expression levels). Each plot included the *p*-value and hazard ratio, providing insights into the prognostic relevance of these genes in glioma survival analysis.

Survival between primary and recurrent glioma presents significant differences, as is shown in Supplementary Figure S1. Therefore, we performed a survival analysis to screen out the key genes that were most related to patient prognoses (Figure 2). Figure 2A represented the results of a LASSO-COX regression analysis conducted to identify key genes associated with the recurrence and primary presence of COVID-19-induced glioma, focusing on differentially expressed genes. Combined with the forest graph shown in Figure 2B, the analysis successfully narrowed down six crucial genes: MYBL2, RBM6, VEPH1, AHNAK2, GNG10, and DUSP14, along with the hazard ratios and confidence intervals for each gene. MYBL2 exhibited a significant hazard ratio of 1.25 (CI: 1.14–1.38, *p* = 3.2 × 10^−6^), indicating its potential role in worse outcomes in high-expression group. RBM6 displayed a protective hazard ratio of 0.59 (CI: 0.45–0.78, *p* = 2.3 × 10^−4^), suggesting improved survival in high-expression group. VEPH1 showed a protective effect with a hazard ratio of 0.90 (CI: 0.85–0.96, *p* = 9.0 × 10^−4^). AHNAK2 presented a hazard ratio of 1.17 (CI: 1.05–1.29, *p* = 3.3 × 10^−3^). GNG10 recorded a hazard ratio of 1.48 (CI: 1.10–2.00, *p* = 0.01). DUSP14 indicates a hazard ratio of 1.25 (CI: 1.02–1.53, *p* = 0.03). The Kaplan–Meier survival curves in Figure 2C–H highlight the survival differences between low and high-expression groups of these genes, emphasizing their potential prognostic significance in managing COVID-19 infection-induced glioma. The tendency and significance in Kaplan–Meier survival curves for the genes MYBL2, RBM6, VEPH1, AHNAK2, GNG10, and DUSP14 were in consistence with the forest graph in Figure 2B.

After the selection of key genes to predict the prognosis of COVID-19 infection induced glioma, we also performed univariate and multivariate Cox proportional hazard analyses to further confirm the significance of these genes. The data in Table 1 summarizes the results of both univariate and multivariate Cox proportional hazards analyses for six genes. Under univariate analysis: AHNAK2 had a hazard ratio (HR) of 1.061 (95% CI: 1.039–1.083) with a *p*-value < 0.001, indicating a significant association with the outcome. DUSP14 showed an HR of 1.096 (95% CI: 1.074–1.118) and a *p*-value < 0.001, suggesting a significant risk factor. GNG10 presented an HR of 1.031 (95% CI: 1.023–1.039), also significant with a *p*-value < 0.001. MYBL2’s HR was 1.032 (95% CI: 1.023–1.041), with a *p*-value < 0.001, indicating significance. RBM6 had an HR of 0.991 (95% CI: 0.988–0.994) and a *p*-value < 0.001, indicating a protective factor. VEPH1 displayed an HR of 0.866 (95% CI: 0.824–0.91) with a *p*-value < 0.001, also suggesting a protective role. Under the multivariate analysis: AHNAK2’s HR became 1.028 (95% CI: 1.004–1.053) with a *p*-value of 0.024, remaining significant. DUSP14 had a reduced HR of 1.047 (95% CI: 1.022–1.073), with a persistent significance at *p*-value < 0.001. GNG10 showed an HR of 1.019 (95% CI: 1.011–1.027) with a *p*-value < 0.001, retaining significance. MYBL2 maintained significance with an HR of 1.024 (95% CI: 1.015–1.033) and a *p*-value < 0.001. RBM6’s HR was slightly adjusted to 0.992 (95% CI: 0.988–0.995) with a significant *p*-value < 0.001. VEPH1′s HR was 0.906 (95% CI: 0.862–0.953) with a *p*-value < 0.001, continuing to suggest a protective effect.

Overall, all six genes showed significant associations with the outcome in both univariate and multivariate analyses, though the strength and direction of the associations vary slightly between models.

### 2.3. Analysis of the Immunomodulatory Role of Vitamin D in COVID-19 Infection-Induced Glioma

**Figure 3:**
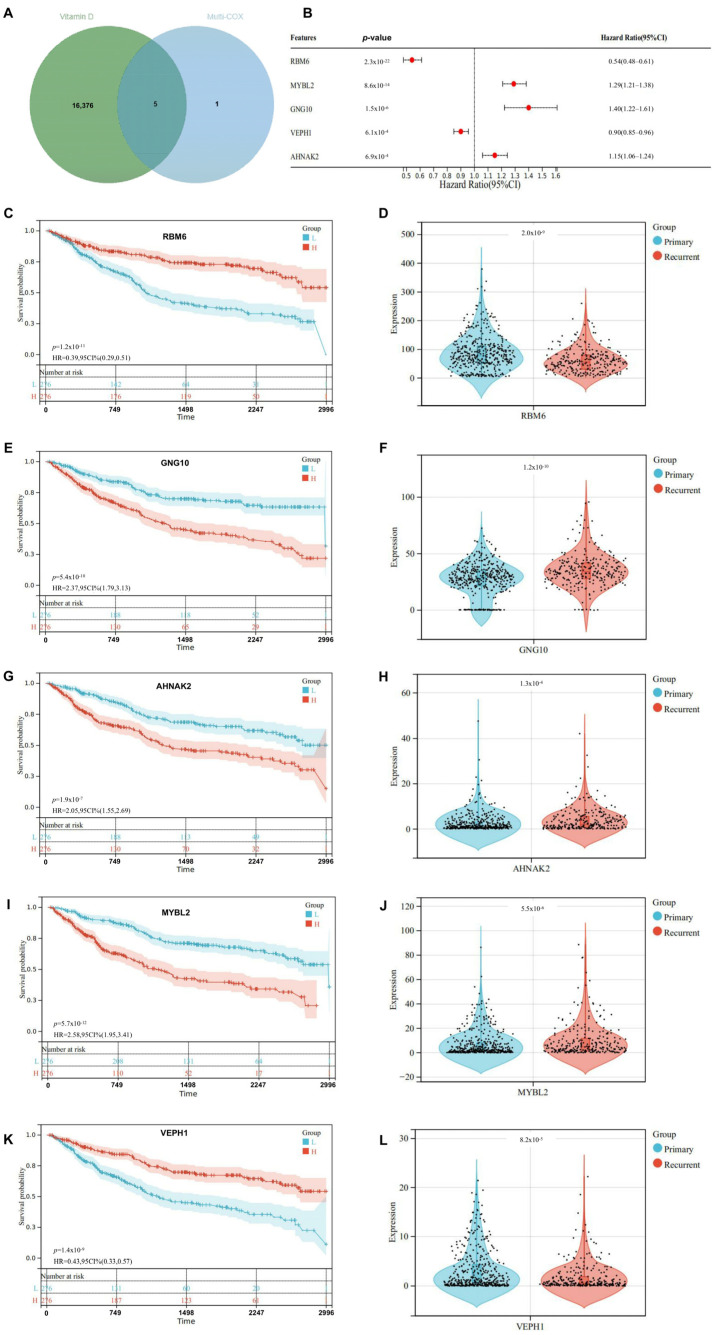
This figure illustrated the selection and analysis of significant differential genes related to COVID-19 induced glioma targeted by vitamin D. (**A**) The Venn diagram displayed the intersection of genes identified through multivariate analysis (blue circle) and vitamin D target genes (green circle). Five genes were found at the intersection, indicating their potential relevance as target genes influenced by both factors. (**B**) This plot presented the hazard ratios (HR) and 95% confidence intervals (CI) for the intersecting genes. Each gene was accompanied by its *p*-value and HR, indicating its statistical significance and risk contribution. Red dots represented hazard ratios, with the horizontal line depicting the confidence interval. (**C**) The survival analysis for RBM6 demonstrated a significant difference between high expression (**H**) and low expression (**L**) groups (*p* = 1.2 × 10^−11^). The hazard ratio was 0.39 (95% CI: 0.29–0.51), indicating better survival in high expression group as a protective factor. (**D**) Violin plots of gene expression for RBM6 depicted the distribution of RBM6 expression levels in primary versus recurrent samples. A statistically significant difference (*p* = 2.0 × 10^−9^) was observed. (**E**) The Kaplan–Meier plot for GNG10 showed a significant survival difference (*p* = 5.4 × 10^−10^), with a hazard ratio of 2.37 (95% CI: 1.79–3.13), suggesting worse survival associated with increased expression. (**F**) GNG10 expression in primary and recurrent samples was significantly different (*p* = 1.2 × 10^−10^), with the plot showing increased expression in recurrent samples. (**G**) Survival curves for AHNAK2 indicated a significant disparity (*p* = 1.9 × 10^−7^) between groups, with a hazard ratio of 2.05 (95% CI: 1.55–2.69). (**H**) AHNAK2 expression showed a significant difference between the two groups was noted (*p* = 1.3 × 10^−4^). (**I**) The survival for MYBL2 was better in the low expression group (*p* = 5.7 × 10^−12^), with a hazard ratio of 2.58 (95% CI: 1.95–3.41). (**J**) MYBL2 expression levels significantly varied between primary and recurrent samples (*p* = 5.5 × 10^−6^). (**K**) The survival curves of VEPH1 showed a better survival for the high expression group (*p* = 1.4 × 10^−9^) with a hazard ratio of 0.43 (95% CI: 0.33–0.57). (**L**) Violin plots showed significant differential expression of VEPH1 in primary versus recurrent samples (*p* = 8.2 × 10^−5^).

Figure 3 provided a detailed analysis of genes identified at the intersection of vitamin D-related genes and genes screened out from the multivariate Cox proportional hazards analysis, further examining their survival relevance and expression in recurrent versus primary glioma groups. The Venn diagram in Figure 3A highlighted five shared genes between vitamin D-related and multivariate genetic screens. The forest plot in Figure 3B explained the hazard ratios of genes in the intersection. RBM6 and VEPH1 were indicated as protective factors with hazard ratios below 1. MYBL2, GNG10, and AHNAK2 showed hazard ratios greater than 1, suggesting they were risk factors for poorer outcomes. The survival curves in Figure 3C,E,G,I,K showed the survival of patients based on the classification of gene expression groups. RBM6 and VEPH1 exhibited better survival in high gene expression groups, with significant *p*-values (RBM6 *p* = 1.2 × 10^−11^, VEPH1 *p* = 1.4 × 10^−9^). MYBL2, GNG10, and AHNAK2 were found to be associated with better survival in low expression groups (e.g., MYBL2 *p* = 5.7 × 10^−12^). Expression of these genes shown in violin/bar plots (Figure 3D,F,H,J,L) compared the gene expression of these genes in primary and recurrent groups. RBM6 and VEPH1 presented higher expression in primary gliomas, with RBM6 *p* = 2.0 × 10^−9^ and VEPH1 *p* = 8.2 × 10^−5^, indicating a significant potential protective role. MYBL2, GNG10, and AHNAK2 exhibited an elevated gene expression in recurrent gliomas, suggesting involvement in cancer progression (e.g., MYBL2 *p* = 5.5 × 10^−6^). This analysis underscored the prognostic value of these genes, revealing distinct survival impacts and expression patterns that correlated with glioma progression and recurrence.

### 2.4. Enrichment Analysis of Common Genes in COVID-19 Induced Glioblastoma Targeted by Vitamin D

**Figure 4:**
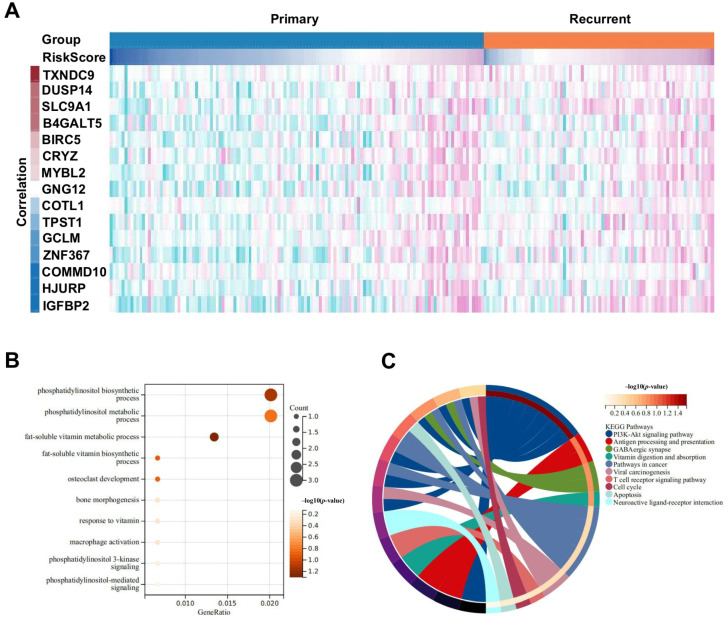
Enrichment analysis of common genes in COVID-19-induced glioblastoma targeted by vitamin D. (**A**) Pearson correlation heatmap displaying the relationship between a vitamin D-related gene risk score and other genes. Each cell represented the correlation coefficient, with color indicating strength and direction: red for positive correlations and blue for negative correlations. The bar above the heatmap categorizes samples into two distinct groups. (**B**) Bubble plot of significantly enriched biological processes and KEGG pathways associated with genes correlated to the vitamin D risk score. Bubbles indicate the number of genes involved (count), and the significance level (*p*-value) is shown by color intensity, with darker shades representing higher significance. (**C**) Chord diagram illustrating interconnected KEGG pathways enriched among the significant genes. Colored bands demonstrate shared genes between pathways, highlighting functional links and interactions. The legend matched pathways to corresponding bands in the diagram.

Figure 4 presented an integrated analysis utilizing a multivariate risk score derived from specific genes associated with COVID-19 and glioma. The heatmap in Figure 4A at the top illustrated the Pearson correlation among the intersecting genes, with the color gradient indicating varied correlation levels. Significant differential expressed genes identified through correlation analysis were subjected to enrichment analysis, focusing on biological processes and KEGG signaling pathways. The dot plot in Figure 4B on the bottom left highlighted key enriched pathways, particularly emphasizing the PI3K-AKT signaling pathways, which are crucial for their role in cellular processes such as growth, metabolism, and survival. The size and color gradient of the dots represented gene count and significance of enrichment, respectively. The circular diagram in Figure 4C visualized the network of interactions in these pathways, underlining the centrality of the PI3K-AKT pathway in modulating responses relevant to COVID-19-induced glioma development. This emphasized the potential of targeting the PI3K-AKT pathway for therapeutic interventions.

### 2.5. Molecular Docking of These Multiple Genes Targeted by Vitamin D in COVID-19 Infection-Induced Glioma

**Figure 5:**
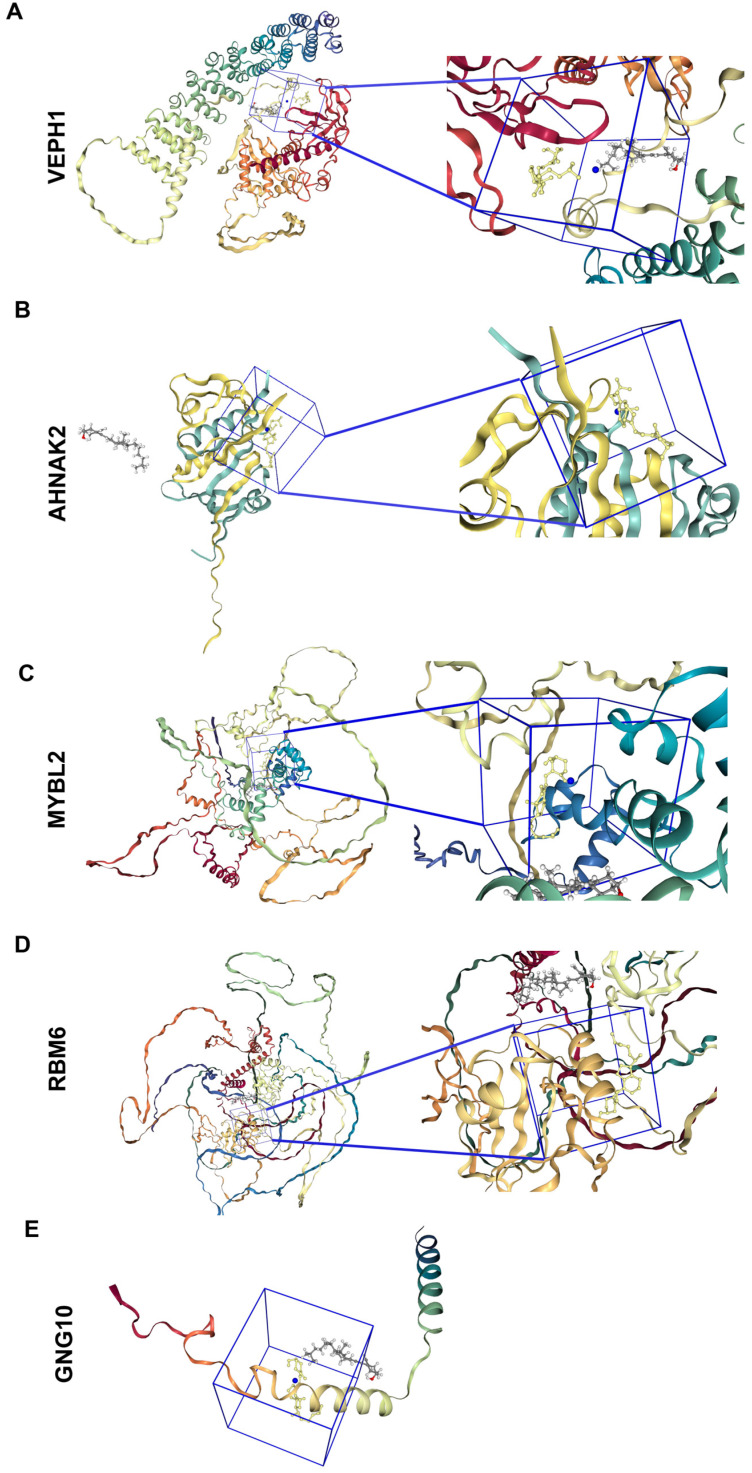
Molecular docking of multiple genes. (**A**) Overall structure of VEPH1 with vitamin D. The protein was shown in ribbon representation, colored by secondary structure elements. The ligand was depicted in a ball-and-stick model. The inset highlighted the ligand binding site, with key interactions involving specific amino acid residues annotated. (**B**) Detailed view of AHNAK2 in complex with vitamin D. The protein’s structural elements were color-coded. The ligand was shown within its binding pocket. The inset provided a close-up view of the interaction site, indicating important residues contributing to binding. (**C**) Structure of MYBL2 and its interacting ligand vitamin D. The protein ribbon diagram illustrated different structural domains. The ligand was visualized in its binding site. The inset detailed specific interaction points between the ligand and protein residues. (**D**) Representation of RBM6 with an associated ligand vitamin D. The entire protein structure was color-coded by secondary structures. The ligand occupied its binding site, shown in closer detail in the inset, where critical binding interactions were marked. (**E**) Overall structure of GNG10 with vitamin D. The protein was shown in ribbon representation, colored by secondary structure elements. The ligand was depicted in a ball-and-stick model. The inset highlighted the ligand binding site, with key interactions involving specific amino acid residues annotated.

The molecular docking analysis depicted in Figure 5 presented the interaction between vitamin D and five key proteins: VEPH1, AHNAK2, GNG10, MYBL2, and RBM6. Each protein structure was shown with a close-up view of the docking site where vitamin D was bound. The docking scores in Table 2, measured in kJ/mol, indicated the binding possibility between vitamin D and each protein. AHNAK2 exhibited the most favorable interaction, with a docking score of −6.8 kJ/mol, suggesting the greatest binding possibility among the proteins analyzed. This was closely followed by VEPH1, MYBL2, and RBM6, all with scores of −6.6 kJ/mol. GNG10 showed the least favorable interaction, with a docking score of −4.7 kJ/mol, indicating a comparatively weaker binding possibility compared to the critical value of −5 kJ/mol for possible binding interactions [18]. These results provided insights into the potential interactions between vitamin D and these proteins, suggesting a possible biological relevance that may warrant further investigation.

### 2.6. Single Cell RNA Sequencing Analysis of Key Genes Involved in Vitamin D Targeted COVID-19 Infection-Induced Glioma

**Figure 6:**
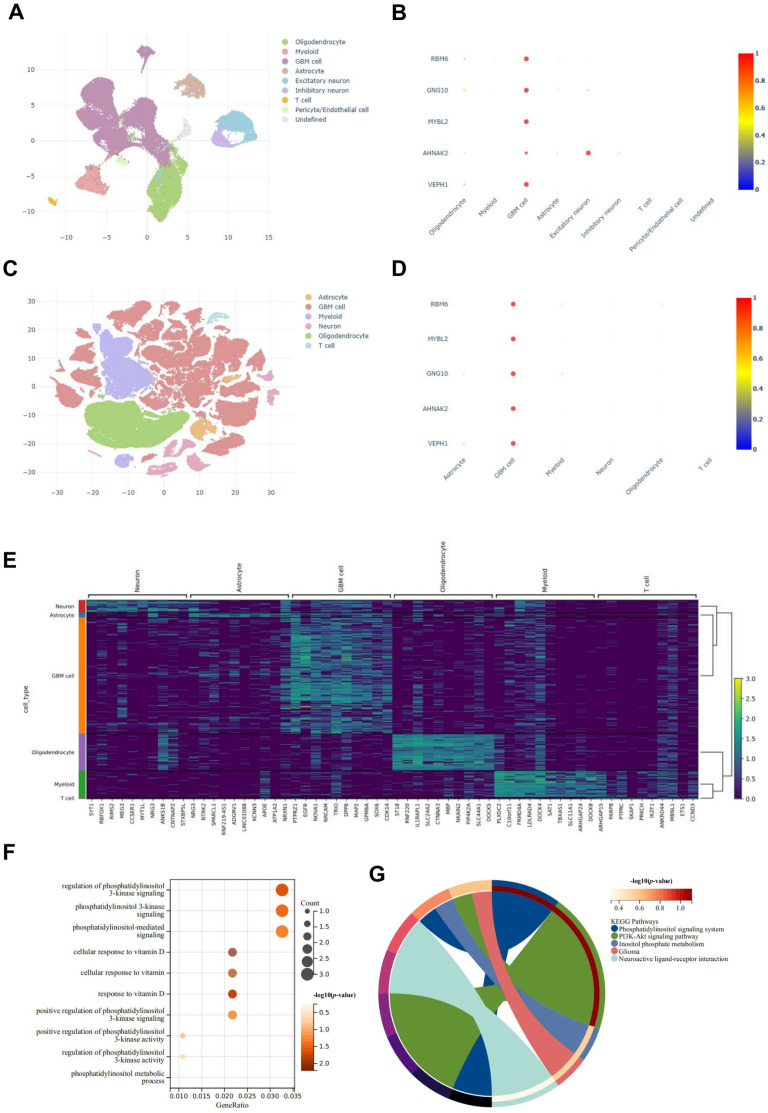
Single-cell RNA sequencing analysis of key genes. (**A**) UMAP plot showing the clustering of cells into distinct types, including oligodendrocytes, myeloid, GBM cells, astrocytes, excitatory neurons, inhibitory neurons, T cells, pericytes/endothelial cells, and undefined cells, based on their expression profiles. (**B**) Dot plot displaying the intersection of genes RBM6, GNG10, MYBL2, AHNAK2, and VEPH1 across different cell types. The color intensity and size of the dots represented the level of gene expression. (**C**) UMAP visualization of a combined dataset highlighting the separation into astrocytes, GBM cells, myeloid cells, neurons, oligodendrocytes, and T cell populations. (**D**) Dot plot focusing on genes intersecting with RBM6, MYBL2, GNG10, AHNAK2, and VEPH1 cell categories, with dot color and size indicating expression magnitude. (**E**) Heatmap illustrating expression patterns of specific genes across diverse cell types. The color gradient intensity depicts the expression levels, with a dendrogram showing clustering based on similarity. (**F**) Bubble plot representing enriched pathways from gene ontology analysis related to PI3K-AKT pathways and vitamin D. The size of the bubbles indicates the number of genes, and the color shows the statistical significance (*p*-value). (**G**) Circular diagram representing connections in KEGG pathways, emphasizing those such as the phosphatidylinositol signaling system, PI3K-Akt signaling, inositol phosphate metabolism, glioma and neuroactive ligand-receptor interaction. The bands’ width and color signify the pathways’ importance and interaction categories.

The image in Figure 6 illustrated a detailed analysis of single-cell datasets, focusing on key genes predominantly enriched in glioma cells. Figure 6A,C showed cell clustering, revealing the distribution of various cell types, including glioblastoma (GBM) cells. Figure 6B,D depicted dot plots highlighting significant genes and their expression levels across different cell types. Figure 6E visualized the expression profile of significant genes in different cell types, emphasizing their variability across different cells. Enrichment analysis of the significant genes was presented in Figure 6F,G. The biological process enrichment (Figure 6F) included terms related to both cellular response to vitamin D and the processes associated with phosphatidylinositol 3-kinase mediated pathways, and the KEGG pathway analysis (Figure 6G) emphasized the involvement in key signaling pathways such as the PI3K-Akt signaling pathway. Importantly, the enrichment of the PI3K-AKT signaling pathway was underscored, suggesting its pivotal role in the biology of glioma cells. This pathway is crucial in regulating cell growth, survival, and metabolism, making it a potential target for therapeutic interventions in gliomas.

## 3. Discussion

This study provided a comprehensive exploration of the molecular interplay between glioma and COVID-19, highlighting the critical role of the PI3K-Akt signaling pathway and the potential modulatory effects of vitamin D. Through integrated bioinformatics analyses, we identified six prognostically significant genes—MYBL2, RBM6, VEPH1, AHNAK2, GNG10, and DUSP14—that are differentially expressed in both glioma and COVID-19 contexts. These findings underscored the intricate molecular connections between cancer progression and viral infection, particularly in the immunosuppressive environment characteristic of glioma patients.

Central to our findings is the PI3K-Akt signaling pathway, which emerged as a pivotal link between glioma pathogenesis and COVID-19-induced molecular alterations. The enrichment analyses consistently highlighted this pathway, corroborating the existing literature that underscores its involvement in cellular processes such as growth, proliferation, and survival in cancer. In the context of viral infections, the PI3K-Akt pathway is known to facilitate viral replication [19] and modulate host immune responses [20]. The convergence of these roles suggests that dysregulation of PI3K-Akt may exacerbate glioma progression during COVID-19 infection, potentially contributing to poorer clinical outcomes [21]. Targeting this pathway could, therefore, represent a strategic approach to mitigate the adverse effects of COVID-19 on glioma patients [22,23].

The identification of MYBL2, RBM6, VEPH1, AHNAK2, GNG10, and DUSP14 as key genes offers valuable insights into the molecular mechanisms underpinning glioma progression and its interaction with COVID-19. MYBL2 (a modulator of MAPK signaling by directly regulating transcription of the gene encoding the negative modulator SPRY2) [24] and AHNAK2 (an activator of the NF-κB pathway) [25], both associated with increased hazard ratios, may serve as potential biomarkers for aggressive glioma phenotypes [26], while RBM6 (a promotor of homologous recombination repair of double-strand breaks and modulator of sensitivity to chemotherapeutic drugs) [27] and VEPH1 (a regulator of the Hippo-YAP signaling pathway) [28], exhibiting protective effects, could be explored as therapeutic targets to enhance patient prognosis. GNG10 (G-protein subunit gamma 10) and DUSP14 (dual specificity phosphatase 14) further contribute to the complex regulatory network, with their roles warranting deeper investigation. The gene MYBL2 is mainly associated with the regulation of cell cycle and cell proliferation processes. This gene is responsible for cell growth and cell differentiation and it is widely expressed in many types of the cells [29]. The gene RBM6 is a type of RNA binding protein containing a RNA binding motif and ribonucleoprotein motif. Usually, this gene is suppressive in the carcinogenic process. The dysregulation of RBM6 gene expression might be associated with cancer development [30]. The VEPH1 is an intracellular adaptor protein which plays important roles in the development of human organs and neural systems. The dysregulation of this gene is associated with the development of many types of cancers, such as ovarian cancer, lung cancer, and liver cancer. This gene exerts key tumor suppressing functions through many signaling pathways, for instance, the AKT signaling pathway [31]. AHNAK2 belongs to the AHNAK gene family, which contains AHNAK2 and AHNAK. This gene is involved in many biological processes such as calcium channel modulation and membrane repair. Research linking this gene family with cancers has been rapidly increasing in recent years. Usually the abnormal expression of this gene is a key regulator in progression of tumors through pathways such as the ERK, MAPK, Wnt, and MEK signaling pathways, and it promotes epithelial-mesenchymal transition [32]. The GNG10 protein participates in G-protein coupled receptor signal transduction, cell proliferation, and differentiation [33]. DUSP14 participates in the inflammatory process and oxidative stress [34]. Notably, the differential expression of these genes in primary versus recurrent gliomas, as revealed by single-cell RNA sequencing, highlights their involvement in glioma recurrence and resistance to therapy, thereby emphasizing their potential as targets for intervention.

Vitamin D’s immunomodulatory and anti-inflammatory properties emerge as significant in the context of glioma and COVID-19. Our molecular docking studies revealed favorable binding affinities between vitamin D and several of the identified key proteins, particularly AHNAK2 and VEPH1. This suggested that vitamin D supplementation could potentially influence the activity of these proteins, thereby modulating the PI3K-Akt pathway and attenuating the immunosuppressive environment in glioma patients. Given the protective associations observed with RBM6 and VEPH1 expression, vitamin D may enhance their beneficial effects, offering a synergistic approach to improve patient outcomes amidst the challenges posed by COVID-19.

The single-cell RNA sequencing analysis provided spatial and functional context to the expression patterns of the key genes within glioma tissues. The enrichment of the PI3K-Akt pathway in glioma cells, particularly in recurrent tumors, aligns with its established role in promoting tumorigenesis and therapy resistance [35]. This granular insight underscores the heterogeneity of glioma at the cellular level and the necessity for targeted therapies that address specific molecular alterations. By elucidating the cellular distribution and functional dynamics of the identified genes, this study paves the way for more personalized therapeutic strategies that consider both the genetic landscape of the tumor and the modulatory impact of concurrent viral infections.

Despite the significant insights garnered, this study is not without limitations. The reliance on publicly available datasets may introduce biases related to sample selection and data quality. Additionally, while bioinformatics analyses provide valuable predictions, experimental validation is essential to confirm the functional roles of the identified genes and the therapeutic potential of Vitamin D interactions. Future studies should incorporate in vitro and in vivo experiments to elucidate the mechanistic pathways and assess the efficacy of PI3K-Akt inhibitors and vitamin D supplementation in improving prognosis for glioma patients, particularly those affected by COVID-19.

In conclusion, our findings illuminated the molecular association between glioma and COVID-19, emphasizing the PI3K-Akt signaling pathway and vitamin D-mediated immunomodulation as critical factors influencing disease progression and patient outcomes. By identifying key prognostic genes and elucidating their interactions with Vitamin D, this study contributes to a deeper understanding of the shared pathogenic mechanisms and opens avenues for developing targeted interventions. These insights are particularly pertinent in the ongoing efforts to enhance therapeutic strategies and improve the resilience of glioma patients in the face of concurrent viral infections.

## 4. Materials and Methods

### 4.1. Omics and Clinical Data Acquisition

RNA-seq data and paired clinical information for glioma cancer samples were obtained from the China Glioma Genome Atlas (CGGA) database (http://www.cgga.org.cn/) (accessed on 10 October 2024) [36]. A total of 552 patients with complete survival information were included in this study. Additionally, two single-cell RNA sequencing (scRNA-seq) datasets of glioma cancer, SCDS0000043 and SCDS0000041, were sourced from The China National GeneBank (CNGB) database (https://db.cngb.org/) (accessed on 10 October 2024) [37].

The drug targets of vitamin D were downloaded from the BATMAN-TCM Database (http://bionet.ncpsb.org.cn/batman-tcm/index.php) (accessed on 10 October 2024) [38]. PI3K/AkT signaling pathway-associated genes were collected from the Gene Set Enrichment Analysis (GSEA) database (https://www.gsea-msigdb.org/gsea/index.jsp) (accessed on 10 October 2024) [39,40].

### 4.2. Expression Analysis of Key Genes Related to COVID-19 Induced Glioma

The differential gene expression analyses for both COVID-19 infection and glioma were performed using the Limma algorithm through Rstudio software (version 4.3.1). Then, common significant differentially expressed genes in these diseases were collected.

### 4.3. Prognostic Model Construction

A prognostic model for COVID-19-induced glioma and COVID-19-induced glioma under vitamin D treatment was constructed using least absolute shrinkage and selection operator (LASSO) Cox regression, Kaplan–Meier (KM) survival curves, and multivariate survival regression. These methods also facilitated the identification of key genes mediating glioblastoma recurrence induced by COVID-19 infection targeted by vitamin D. All analyses were performed using RStudio software (version 4.3.1).

### 4.4. Cox Proportional Hazards Model

The Cox proportional hazards model was established to evaluate the statistical correlation between hazard factors and glioma recurrence. Specifically, variables including key genes screened from the prognostic model construction were analyzed using univariate Cox regression via IBM SPSS Statistics software 30.0.0. Genes significantly associated with tumor recurrence were subsequently included in a multivariate Cox regression analysis to identify the primary genes influencing glioma recurrence.

### 4.5. Molecular Docking

Molecular docking was performed to predict the binding modes and affinities between vitamin D and key proteins in glioblastoma using the online platform EZCADD (http://dxulab.pharmacy.isu.edu/curry/ezCADD/main.html) (accessed on 18 October 2024). The spatial structures of the MYBL2 (P10244), RBM6 (P78332), VEPH1 (Q14D04), AHNAK2 (4CN0), and GNG10 (P50151) proteins and vitamin D (PubChem CID: 5280795) were obtained from the Protein Data Bank (https://www.rcsb.org/) (accessed on 18 October 2024), Uniprot (https://www.uniprot.org/) (accessed on 18 October 2024) and PubChem Database (https://pubchem.ncbi.nlm.nih.gov/) (accessed on 18 October 2024) and then uploaded into EZCADD to determine the virtual binding possibility of vitamin D to form the vitamin D–protein complex. The spatial structures of MYBL2, RBM6, VEPH1, and GNG10 were predicted using the AlphaFold algorithm. The potential binding pocket with the largest volume was selected for vitamin D docking. Docking scores were calculated, with lower scores indicating stronger binding possibility. A docking score below −5 kcal/mol was considered significant [18].

### 4.6. Pearson Correlation

Pearson correlation analysis was conducted using RStudio (version 4.3.1) to identify genes associated with prognostic model genes in glioma. Correlation coefficients ranged from −1 to 1, indicating the strength and direction of the association. Positive correlations (values > 0) and negative correlations (values < 0) were identified, and statistical significance was assessed to filter for genuinely prognostic-correlated genes.

### 4.7. Enrichment Analysis

Gene annotations were accessed through the Kyoto Encyclopedia of Genes and Genomes (KEGG) database (https://www.kegg.jp/kegg/rest/keggapi.html) (accessed on 10 October 2024). The “ClusterProfiler” R package (R 4.4.0) was utilized to perform enrichment analysis on genes involved in the therapeutic effects of vitamin D on COVID-19 infection induced glioma recurrence. Analyses included Gene Ontology (GO), KEGG pathway enrichment, and Gene Set Enrichment Analysis (GSEA).

### 4.8. scRNA-Seq Analysis

The Seurat R package (4.4.0) was used to perform scRNA-seq analysis on datasets SCDS0000043 and SCDS0000041 in RStudio, utilizing the “FindAllMarkers” function. Genes with absolute Log2 fold change values exceeding 0.25 were identified as differentially expressed and subjected to further enrichment analysis. Additionally, the “CellChat” R package (R 4.4.0) was employed to elucidate cell–cell communication among various immune cells at single-cell resolution.

### 4.9. Statistical Analysis

A Student’s *t*-test was employed to assess differences between two groups. The Pearson correlation coefficient identified genes significantly correlated with prognostic model genes. Clinical factors significantly linked to the key gene were selected using the Chi-squared test. Statistical significance was established at a *p*-value < 0.05.

## 5. Conclusions

This study explores the intricate molecular interactions between glioma and COVID-19, emphasizing the pivotal role of the PI3K-Akt signaling pathway and the immunomodulatory effects of vitamin D. Through extensive bioinformatics analyses—including enrichment, survival, and molecular docking assessments—we identified six key prognostic genes (MYBL2, RBM6, VEPH1, AHNAK2, GNG10, and DUSP14) that are differentially expressed in both glioma and COVID-19 contexts. After intersecting with the targets of Vitamin D, five genes (MYBL2, RBM6, VEPH1, AHNAK2, and GNG10) were screened out. These genes are significantly involved in the PI3K-Akt pathway, highlighting its central role in tumor progression and viral response. Molecular docking results reveal that vitamin D can effectively bind to several of these proteins, especially AHNAK2 and VEPH1, suggesting a potential therapeutic mechanism by which vitamin D supplementation may improve patient prognosis. Additionally, single-cell RNA sequencing provided insights into the spatial and functional expression patterns of these genes within glioma tissues, distinguishing their roles in primary versus recurrent tumors. The convergence of the PI3K-Akt pathway in both glioma progression and COVID-19 infection underscores its potential as a strategic therapeutic target. Vitamin D’s ability to modulate this pathway and interact with critical prognostic genes presents a promising strategy for enhancing outcomes in glioma patients affected by COVID-19. However, the study is limited by its reliance on publicly available datasets and requires experimental validation to confirm the functional roles of the identified genes and the therapeutic potential of vitamin D. Future research should focus on in vitro and in vivo studies to validate these bioinformatics predictions and investigate the efficacy of PI3K-Akt inhibitors and vitamin D supplementation as potential treatments. Overall, our findings advance the understanding of shared molecular mechanisms between glioma and COVID-19, paving the way for targeted therapeutic strategies and personalized medicine approaches to improve the resilience and prognosis of glioma patients amidst viral infections.

## Figures and Tables

**Table 1:** Univariate and multivariate analysis of common genes between glioblastoma and COVID-19.

Genes	Univariate Analysis		Multivariate Analysis	
	Hazard Ratio (95% CI)	*p* Value	Hazard Ratio (95% CI)	*p* Value
AHNAK2	1.061 (1.039–1.083)	<0.001	1.028 (1.004–1.053)	0.024
DUSP14	1.096 (1.074–1.118)	<0.001	1.047 (1.022–1.073)	<0.001
GNG10	1.031 (1.023–1.039)	<0.001	1.019 (1.011–1.027)	<0.001
MYBL2	1.032 (1.023–1.041)	<0.001	1.024 (1.015–1.033)	<0.001
RBM6	0.991 (0.988–0.994)	<0.001	0.992 (0.988–0.995)	<0.001
VEPH1	0.866 (0.824–0.91)	<0.001	0.906 (0.862–0.953)	<0.001

This table presents the univariate and multivariate analysis of common genes associated with both glioblastoma and COVID-19. The hazard ratios (HR) with their respective 95% confidence intervals (CI) are provided for each gene, indicating the strength and direction of the association with the outcome. *p*-values less than 0.05 are considered statistically significant, denoting a meaningful relationship between the gene expression and the diseases.

**Table 2:** Docking scores of vitamin D into key genes in COVID-19-induced glioblastoma.

Genes	Docking Score (kJ/mol)
VEPH1	−6.6
AHNAK2	−6.8
GNG10	−4.7
MYBL2	−6.6
RBM6	−6.6

This table presents the docking scores for the COVID-19 infection-induced glioma genes targeted by vitamin D. Lower docking scores indicate higher binding possibility.

## Data Availability

The original contributions presented in the study are included in the article/Supplementary Materials. Further inquiries can be directed to the corresponding authors.

## Supplementary Materials

The following supporting information can be downloaded at: https://www.mdpi.com/article/10.3390/ijms252312952/s1.
