# Supplementary figures and images for: The Immunomodulatory Effects of Vitamin D on COVID-19 Induced Glioblastoma Recurrence via the PI3K-AKT Signaling Pathway
Source: Int J Mol Sci. 2024 Dec 2;25(23):12952. doi: 10.3390/ijms252312952 (PMC11641820; doi:10.3390/ijms252312952)

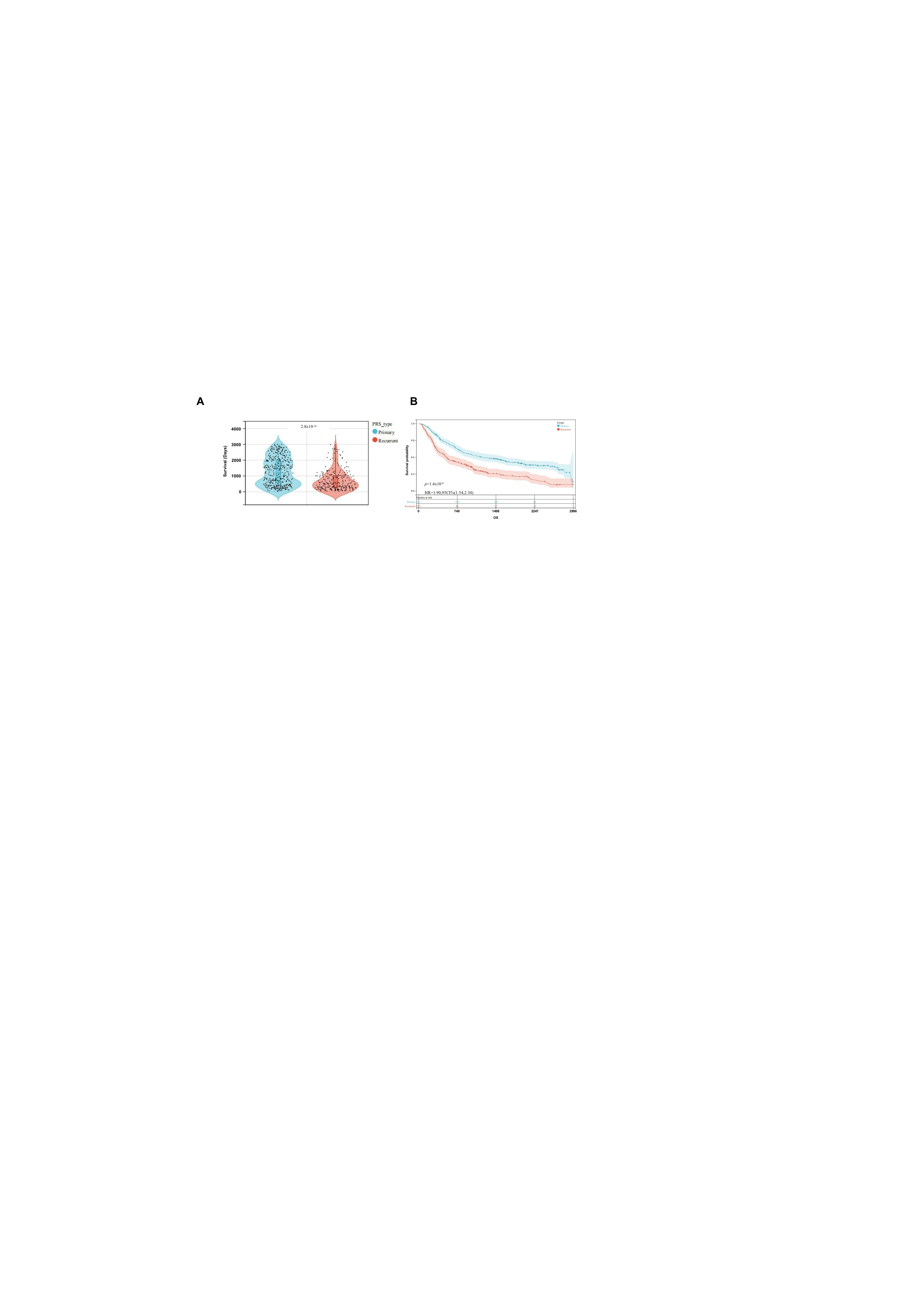

Supplement: Supplementary file 1 [file ijms-25-12952-s001.zip › Supplement Figure/Supplement Figure 1.jpg]
